# Utilizing differences in bTH tolerance between the parents of two-line hybrid rice to improve the purity of hybrid rice seed

**DOI:** 10.3389/fpls.2023.1217893

**Published:** 2023-08-03

**Authors:** Xiuli Zhang, Qing Wang, Guojian Fan, Li Tang, Ye Shao, Bigang Mao, Qiming Lv, Bingran Zhao

**Affiliations:** ^1^ Longping Branch, College of Biology, Hunan University, Changsha, China; ^2^ State Key Laboratory of Hybrid Rice, Hunan Hybrid Rice Research Center, Changsha, China; ^3^ College of Agronomy, Hunan Agricultural University, Changsha, China

**Keywords:** two-line hybrid rice, seed production, HIS1 gene, β-triketone herbicide (bTH), seed soaking

## Abstract

**Introduction:**

Two-line hybrid rice based on Photoperiod/thermo-sensitive genic male sterile (P/TGMS) lines has been developed and applied widely in agriculture due to the freedom in making hybrid combinations, less difficulty in breeding sterile lines, and simpler procedures for breeding and producing hybrid seed. However, there are certain risks associated with hybrid seed production; if the temperature during the P/TGMS fertility-sensitive period is lower than the critical temperature, seed production will fail due to self-pollination. In a previous study, we found that the issue of insufficient purity of two-line hybrid rice seed could be initially addressed by using the difference in tolerance to β-triketone herbicides (bTHs) between the female parent and the hybrid seeds.

**Methods:**

In this study, we further investigated the types of applicable herbicides, application methods, application time, and the effects on physiological and biochemical indexes and yield in rice.

**Results:**

The results showed that this method could be used for hybrid purification by soaking seeds and spraying plants with the bTH benzobicylon (BBC) at safe concentrations in the range of 37.5-112.5 mg/L, and the seeds could be soaked in BBC at a treatment rate of 75.0 mg/L for 36-55 h without significant negative effects. The safe concentration for spraying in the field is 50.0-400.0 mg/L BBC at the three-leaf stage. Unlike BBC, Mesotrione (MST) can only be sprayed to achieve hybrid purification at concentrations between 10.0 and 70.0 mg/L without affecting yield. The three methods of hybrid seed purification can reach 100% efficiency without compromising the nutritional growth and yield of hybrid rice. Moreover, transcriptome sequencing revealed that 299 up-regulated significant differentially expressed genes (DEGs) in the resistant material (Huazhan) poisoned by BBC, were mainly enriched in phenylalanine metabolism and phenylpropanoid biosynthesis pathway, it may eliminate the toxic effects of herbicides through this way.

**Discussion:**

Our study establishes a foundation for the application of the bTH seed purification strategy and the three methods provide an effective mechanism for improving the purity of two-line hybrid rice seeds.

## Introduction

Two-line hybrid rice based on Photoperiod/thermo-sensitive genic male sterile (P/TGMS) lines has been developed and applied widely in agriculture and plays a crucial role in ensuring food security in China (Yuan, 1997; Ma and Yuan, 2015; Mou, 2016; Yuan, 2017). Compared with the three-line system, the superiority of the two-line hybrid rice includes a one-line, dual-use simplified breeding procedure (Yuan, 1990). The fertility of P/TGMS is controlled by the photoperiod and temperature. Different ecological areas where rice is grown require different lower temperature limits for the fertility conversion of P/TGMS; for example, the Yangtze River basin ecological area in China requires an average daily temperature of 23°C, while it is 24°C for the South China (Yuan, 1990; Mou, 2016; Wu et al., 2021). When it comes to long photoperiods and high temperatures, P/TGMS lines are male sterile and can be crossed with restorer lines to produce hybrid seeds. Otherwise, fertility in the female parent will result in self-crossing. The advantages of two-line hybrid rice also include a wide recovery spectrum, many diverse sterile lines, and freedom of choice in making hybrid combinations, which explains why two-line hybrid rice has been developed and applied widely in agriculture. (Yuan, 1977; Yuan, 1990; Yuan, 1997; Ashraf et al., 2020). By 2015, the annual cultivation area of two-line hybrid rice in China reached 4.59 million hm^2^, accounting for 37.8% of the total land area devoted to hybrid rice. At present, two-line hybrid rice has become the main way to utilize heterosis in rice (Yuan, 1990; Ma and Yuan, 2015; Zhou G. et al., 2012; Pak et al., 2021). On the international front, the United States was the first country to make use of China’s hybrid rice strategy. Breeders in the US considered the development of two-line hybrid rice varieties to be of key importance. They used P/TGMS lines introduced from China to cultivate new two-line combinations with high yield and good grain quality that are suitable for the local environment, so as to realize large-scale commercial seed production (Deng, 1998; Hu and Jiang, 2011). However, the maternal parent is susceptible to low-temperatures, which will restore fertility during seed production of two-line hybrid rice. This results in self-pollination which decreases the purity of commercial hybrid seeds, and can even lead to seed production failures. There are many documented cases of large-scale seed production failure due to the low-temperature meteorological risk (Lei et al., 2013; Lei and Chen, 2015). It has been reported that more than 6,700 hm^2^ seed production failed because P/TGMS female parents experienced continuous low-temperature (<23°C) during the fertility-sensitive period in Yancheng, Jiangsu Province, China in 2009, which caused serious economic losses and restricted the sustainable development of two-line hybrid rice (Shi, 1985; Zhou H. et al., 2012; Lei et al., 2014). The safety and security of hybrid seed production is the premise and foundation for the popularization and application of two-line hybrid rice; therefore, it is particularly important to address the issues of insufficient seed purity in seed production.

Reducing the critical sterility-inducing temperature of P/TGMS lines is the key to ensuring the safety of two-line hybrid seed production (Yuan, 1992). However, if some female parental lines restore fertility and self-pollinate, the F_1_ hybrid can be purified by herbicide application. The approach to addressing this issue can be found by screening and identifying sterile lines that are sensitive to a certain herbicide to which their restorer lines are correspondingly resistant. Spraying the herbicide on the F_1_ hybrid seedling fields will selectively kill the herbicide-sensitive offspring derived from self-pollination of the female parent. The hybrid seeds cannot be poisoned due to the dominant resistant genes inherited from the restorer lines, which can ensure the purification of F_1_ hybrid seeds produced in the field (Xiao, 2012). Large-scale commercial seed production of two-line hybrid rice in the United States also benefits from the hybrid seed purification methods. In 2001, the Louisiana State University Agricultural Center (LSU AgCenter, Baton Rouge, LA, USA), introduced imidazolinone (IMI)-resistant restorer rice lines by chemical mutagenesis using ethyl methanesulfonate (EMS). Thus IMI, an inhibitor of acetolactate synthase (ALS), is used to purify two-line seed production extensively in the US (Webster and Masson, 2001; Croughan, 2003; Sudianto et al., 2013). However, imidazolinone herbicides have a long residual period, which can easily damage sensitive crops in crop rotations, and there are many ALS-resistant weeds. At present, 66 weeds are known to carry ALS resistance due to mutation. (Jin et al., 2022). β-triketone herbicides (bTHs) are a class of herbicide that inhibits 4-hydroxyphenyl-pyruvate dioxygenase (HPPD). bTHs have broad spectrum activity against annual grasses and many dicot weeds but are safe on cereal crops and have great application prospects (Ahrens et al., 2013; Ndikuryayo et al., 2017; Lv et al., 2021). The main types of bTHs used in agriculture are mesotrione (MST), sulcotrione (SLT), benzobicylon (BBC), tembotrione (TMT), and tefuryltrione (He et al., 2012; Su and Teng, 2013; Hua, 2015). As a systemic, broad-spectrum selective herbicide, MST can effectively kill broadleaf weeds and many weedy grass species. The half-life of the prodrug component in soil and plants is only about 3 days, making it safe for birds, insects, and the environment (Sun et al., 2013; Su et al., 2017). MST has been applied widely in agriculture due to its low toxicity, low residue levels, and high activity (Bai, 2021; Zhang et al., 2019). As a novel paddy-bleaching herbicide, BBC has been widely applied in puddled-transplanted (PTR) rice and direct seeded rice (DSR) crops because of its superior broad-spectrum activity against annual grasses, broadleaf weeds, low toxicity on *japonica* rice, and high efficacy against paddy weeds resistant to other types of herbicides (Lv et al., 2021). A major advantage is that MST/BBC can be used to improve seed purification in two-line hybrid rice seed production.

At present, there is a lack of relevant research on using herbicides to improve the purity of two-line hybrid seed production. The published studies have mainly relied on transgenic or mutagenic approaches which cannot be generally applied, especially for transgenic crops that have not been commercially planted in China (Zhang et al., 2002; Su et al., 2012; Fan and Zhang, 2018; Pak et al., 2021). In a previous study, we screened a group of bTH-sensitive (*his1-28bp* and *his1-T1510G*) P/TGMS lines by analyzing natural allelic variants of the *HIS1* gene which gives broad-spectrum resistance to bTHs (Lv et al., 2021). The corresponding restorer lines were resistant to β-triketone herbicides, and we initially confirmed that bTHs could be used to ensure the purity of two-line hybrid seed production. In this study, we further investigated the types of applicable herbicides, application methods, application time, and the effects on rice growth and yield, and found that BBC can achieve hybrid seed purity by soaking, because the time required for seed treatment is not critical, and the application is safe. Spray application of both MST and BBC can be used in seed purification. We also confirmed that the growth and yield of hybrids are not significantly affected by bTH application in the appropriate concentration range by analyzing the physiological and biochemical indexes.

## Materials and methods

### Plant materials and genotyping

The rice lines used in the experiments, ‘Huazhan’ (*HIS1*), ‘Lingliangyou942’ (*HIS1/his-28bp*), ‘Xiangling628S’ (*his1-28bp*), and ‘SE21S’ (*his1-T1510G*), were chosen to represent different genotypes in the two-line hybrid rice combinations to test soaking seeds and spraying seedlings with bTHs at different concentrations.

DNA fragments of the *HIS1* gene that contained two natural variable sites were amplified by PCR from rice genomic DNA using 1.1×TsingKe PCR Mix, and the PCR products were sequenced by TsingKe Biotech Company (Changsha, China). The amplification reactions include 1 μl of template DNA (50 ng/μl), 1 μl of each of the forward and reverse primers (10 μM) (CCJ-CF: GTACAACTTGACGATGTATCAAG, CCJ-CR: GTTGAATCTTGCAAATGCAGGAGC), and 17 μl of 1.1×TsingKe PCR Mix. The PCR reaction conditions consisted of a pre-denaturation step at 95°C for 4 min, followed by 35 cycles of 95°C for 20s, 58°C for 20s, and 72°C for 20s, with a final 5 min extension at 72°C. The reaction products were separated by electrophoresis on a 3% agarose gel. Then it was sent to the TsingKe company for sequencing analysis (**Additional Files 1**–**3**).

### Greenhouse experiments

**Figure 1:**
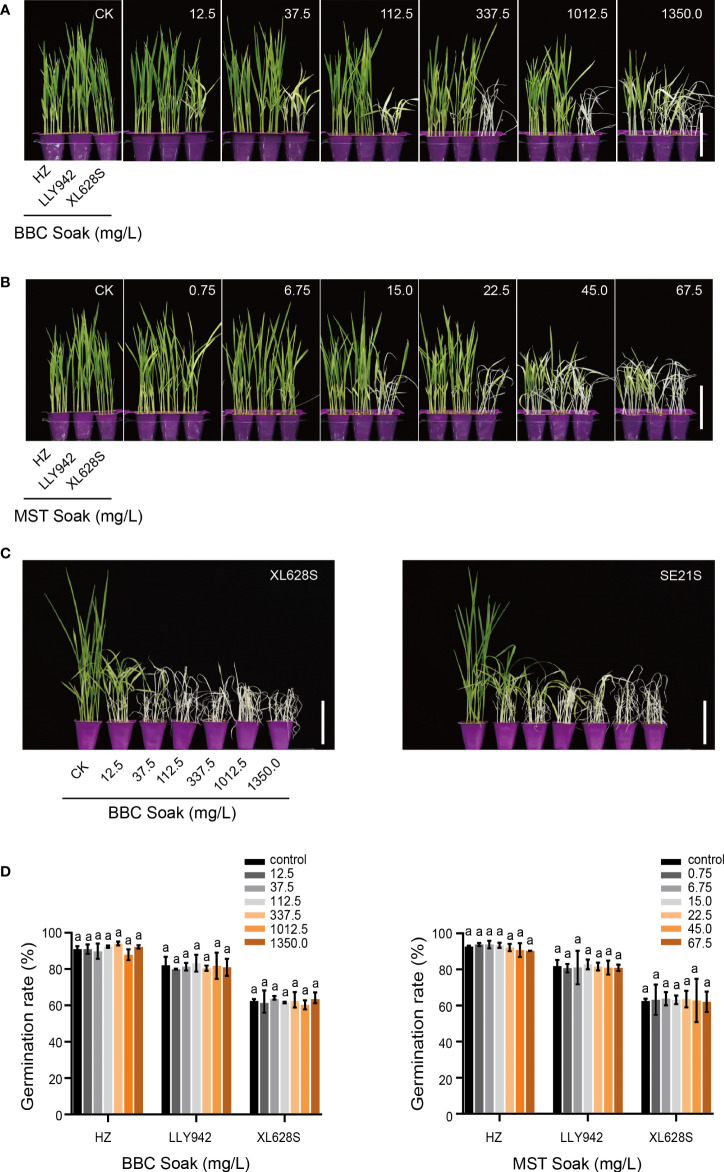
Effects of soaking rice seeds in BBC and MST on purification of two-line hybrid seed production. **(A)** The effects of soaking seeds in different concentrations of benzobicylon (BBC) on the growth of a two-line rice hybrid and the parental lines. **(B)** The effects of soaking seeds in different concentrations of Mesotrione (MST) on the growth of a two-line rice hybrid and the parental lines. **(C)** The effect of soaking seeds in BBC on the growth of two lines carrying natural allelic variants of the *HIS1* gene; XL628S (*his1-28bp*), and SE21S (*his1-T1510G*). **(D)** The effect of soaking seeds of the two-line rice hybrid and the parental lines in different concentrations of BBC and MST on the germination rate. HZ(*HIS1*) is the paternal parent of the two-line hybrid LLY942 (*HIS1/his1-28bp*), and the maternal parent is XL628S (*his1-28bp*). Data are the means ±SD of three biological replicates. The same letters above the bars indicate no significant differences at *p* <0.05 (one-way ANOVA, Tukey’s test). Scale bars = 10 cm.

**Figure 2:**
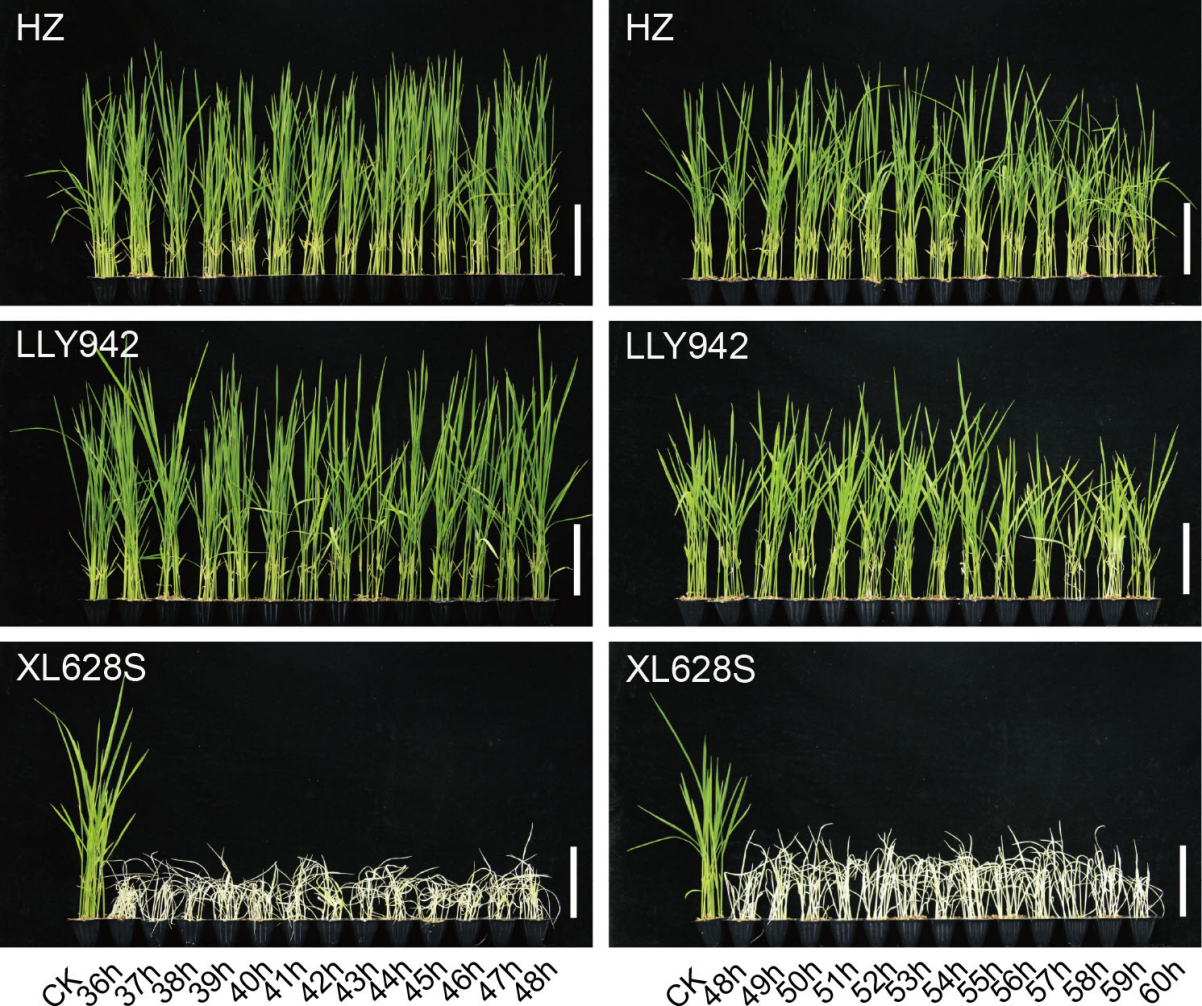
Effect of seed treatment time on the growth of rice seedlings for two-line hybrid seed production. Seeds of the two-line hybrid LLY942 and the parental lines, HZ and XL628S, were soaked in BBC at a concentration of 75.0 mg/L for the indicated times between 36 and 60 hours. Scale bar=10 cm.

**Figure 3:**
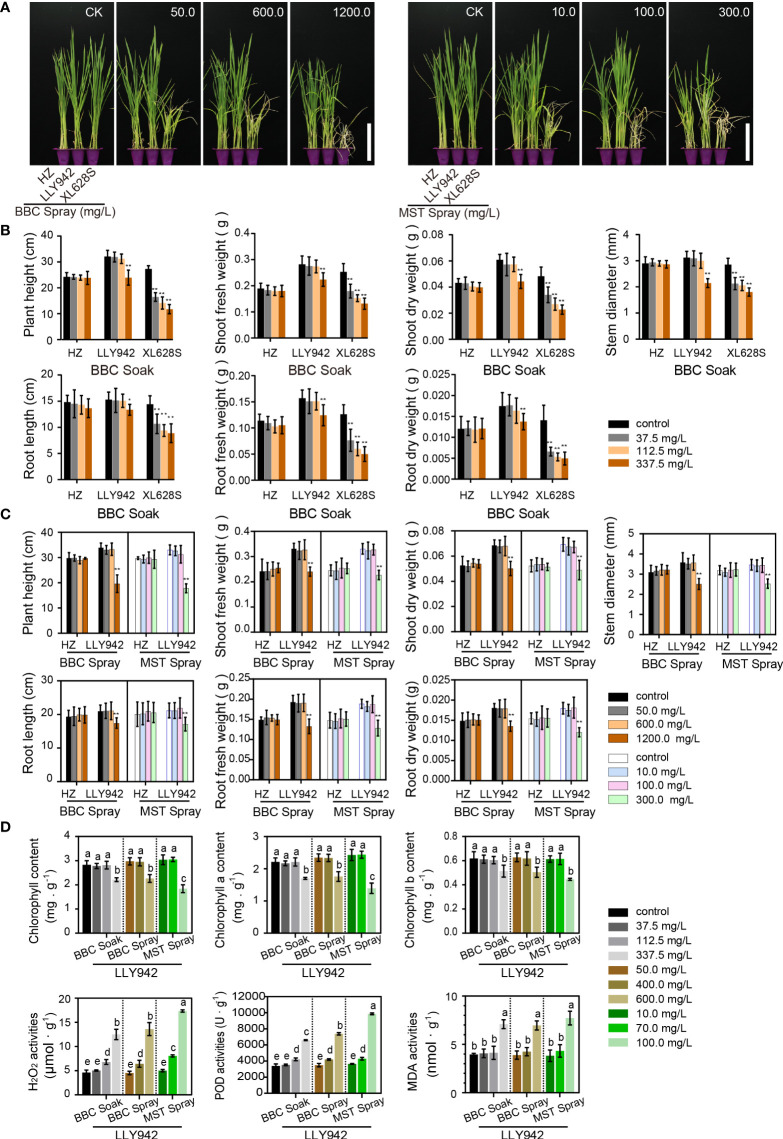
The effects of three different purification methods on the physiological and biochemical indexes of the experimental materials HZ, LLY942, and XL628S. **(A)** Phenotypes of the three lines treated with BBC or MST by spraying at the indicated herbicide concentrations. **(B)** Changes in the growth indexes of the two-line system seedlings grown from seeds treated with different concentrations of BBC. **(C)** Changes in growth indexes of the two-line system materials treated with different concentrations of BBC and MST by spraying. Data are the means ±SD of three biological replicates. In **B** and **C**, asterisks above the bars indicate significant differences between the treatments and controls (*p <0.05, **p <0.01; Student’s t-test). **(D)** Effects of the three purification methods on biochemical activities in the two-line rice hybrid LLY942. Different letters above the bars indicate significant differences at *p* <0.05 (one-way ANOVA, Tukey’s test). Scale bars=10 cm.

Experimental seeds were soaked in tap water or bTH dilutions for 48 h, renewed every 12 h, and allowed to germinate for 15 h in a 37°C incubator. The seeds that germinated well were then sown on the surface of a 4×4 cm vermiculite substrate, and later the seedlings were grown in Yoshida nutrient solution with a pH of 5.5-5.8. Seedlings were grown in the greenhouse with a 14-h light (28°C)/10-h dark (24°C) photoperiod, a light intensity of 700 μmol m^-1^s^-1^, and 60–70% RH (**Figures 1**–**3**). The rice seedlings were sprayed with herbicide to run-off at the three-leaf stage. The plant phenotypes were observed 20 days after sowing.

### RNA isolation and qPCR analysis

Total RNA was isolated from rice leaves using TransZol Up Plus RNA Kit (TransGenER501-01-v2, Beijing, China), and first-strand cDNA was synthesized using TransScript One-Step gDNA Removal and cDNA Synthesis SuperMix (TransGenAT311, Beijing, China). qPCR was performed using ChamQ Universal SYBR qPCR Master Mix (Vazyme Q711, Beijing, China) on a Roche LightCycler 480 II instrument. The housekeeping gene *OsActin* (LOC_Os03g50885) was employed as an internal control. The primers used for quantitative real-time PCR are listed in **Supplementary Table S1**.

### Transcriptome sequencing analyses

HZ, LLY942, XL628S were cultivated in the greenhouse for 10 days. The treatment group was sprayed with 0.4g/L BBC diluent, while the control group was sprayed with tap water. Transcriptome sequencing was performed after 12 hours of treatment. Illumina sequencing was performed by BGI.Tech (http://www.bgitechsolutions.com/). Sequencing data were analyzed and visualized by the use of the free online BGI cloud platform (https://biosys.bgi.com/#/report/login). p-value < 0.05 and |log2FC| >= 1for significantly different expressions.

### Field experiments

**Figure 4:**
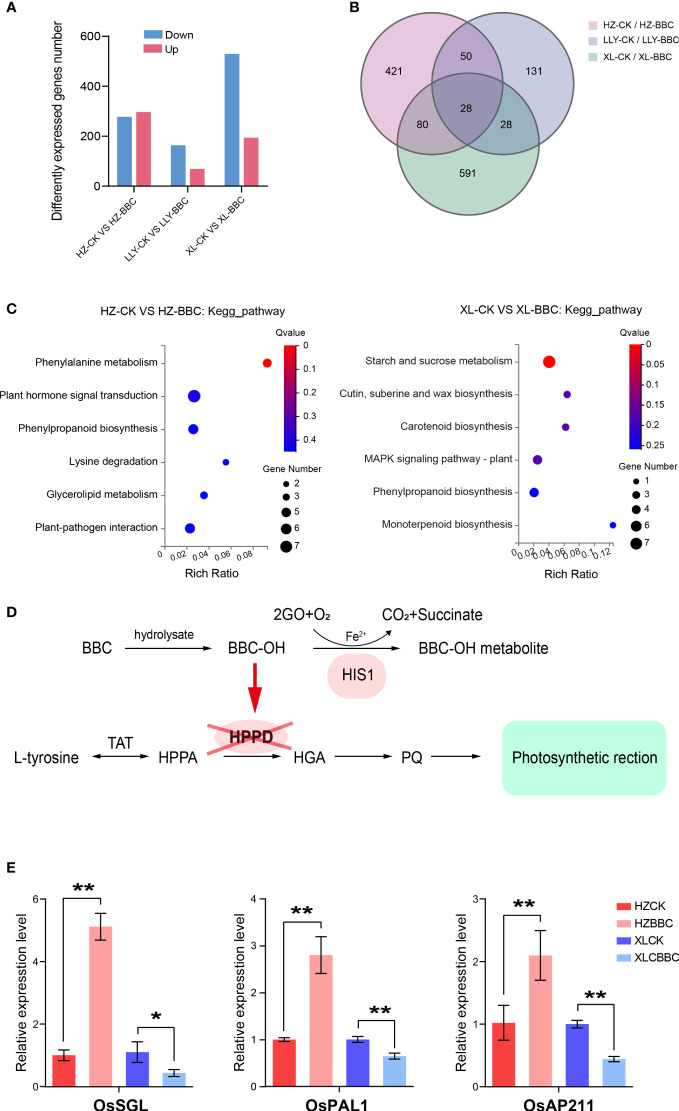
Transcriptome sequencing analysis of HZ, LLY942, XL628S. **(A)** Details of DEGs with a |log2FC| >= 1 (P value< 0.05) in the materials. **(B)** A Venn diagram of the DEGs of HZ, LLY942, XL628S treatment compared to control. **(C)** KEGG pathways that were enriched among HZ and XL628S up-regulated DEGs. **(D)** Metabolic pathway diagram of resistant materials when they are poisoned by BBC herbicides. BBC Benzobicyclon, TAT tyrosine aminotransferase, HPPA hydroxyphenylpyruvic acid, HPPD 4-hydroxyphenylpyruvate dioxygenase, HGA homogentisic acid, PQ plastoquinone. **(E)** Expression level of several genes in phenylpropanoid biosynthesis pathway. Asterisks above the bars indicate significant differences between the treatments and controls (*p <0.05, **p <0.01; Student’s t-test).

The process of soaking the rice seeds in bTHs was consistent with the greenhouse experiment. We prepared a 50 cm×50 cm seedling field, and the germinated seeds were spread evenly on the surface of the seedling field and covered with plastic film. Transplanting was performed using a row spacing of 8 cm×8 cm, and 24 seedlings were planted in each treatment with three experimental replicates. The three-leaf seedlings were sprayed with herbicides at noon on a clear and cloudless day (**Figure 4**).

Five whole rice plants were randomly selected from each experimental spot (the first and last rows were not selected) after the grain was ripe, packed in 20 cm×30 cm mesh bags, and heated for 7 days at 37°C with three experimental replicates. Agronomic traits were assessed in a total of 15 whole rice plants for each experimental material. The agronomic characters were detected with a high-throughput rice digital seed tester (brand: Wuhan Gufeng Optoelectronics, model: YTS-5D).

### Physiological index measurements and analysis

A Mettler ME204 electronic balance was used to determine the fresh weight of stems, leaves, and roots, and a vernier caliper was used to measure the stem diameter. The tissues were heated for 30 min at 120°C and then dried in an oven at 60°C. The related enzyme activities were assayed using Solarbio kits, and the levels of chlorophyll, H_2_O_2_, and MDA, and the POD activity were measured using a 96-well microplate reader (SYNERGY LX, Berten BioTek Instrument Co., Ltd., USA).

Chlorophyll was extracted from plant tissues using a 1:2 v/v mixture of ethanol and acetone. The absorbances of the chlorophyll extracts were measured at two specific wavelengths (645 nm, 663 nm) using a spectrophotometer. The chlorophyll a/b contents were calculated using the formula provided in the Solarbio kits (**Figure 3D**).

H_2_O_2_ reacts with titanium (IV) sulfate to produce peoxotitanium, a compound that can be dissolved in H_2_SO_4_. The determination of H_2_O_2_ content in rice leaves was carried out at a wavelength of 415 nm using the colorimetric method.

Determination of malondialdehyde (MDA) content in rice leaves was determined using the thiobarbituric acid (TAB) method. MDA can react with TBA under acidic, high temperature conditions to form reddish-brown trimethylene. The absorbance of the MDA reaction was measured at three specific wavelengths (600 nm, 532 nm, and 450 nm) using a spectrophotometer. The content of MDA was calculated using the formula provided in the Solarbio kits.

The guaiacol assay was performed to determine the content of peroxidase (POD). POD reacts with guaiacol (2-methoxyphenol) to produce a brown substance with a maximum light absorption at 470 nm. The content of POD was calculated using the formula provided by the Solarbio kits. POD(U/g) = 9800*ΔA/W


W:   Weight (g)

### Chemicals

The experimental herbicide benzobicyclon (BBC) (named “Cong Feng”, 25% effective content suspension concentrate) was obtained from SDS Biotech (Tokyo, Japan) and the Mesotrione herbicide (15% effective content suspension concentrate) is produced by Zhongshan, Anhui Chemical Company.

## Results

### BBC seed treatment can effectively improve seed purity in two-line hybrid rice

Herbicide spraying can kill the female parent without affecting normal growth in the hybrids, but it needs to be done in the field after sowing, which is time-consuming and increases labor costs. To determine whether treating seeds with bTHs before sowing can achieve seed purification, we soaked seeds of ‘Huazhan’ (*HIS1*) (**Additional File 1**), the paternal parent of the two-line hybrid, the two-line rice hybrid ‘Lingliangyou942’ (*HIS1/his1-28bp*), and the maternal parent ‘Xiangling628S’ (*his1-28bp*) (**Additional File 2**), at 37°C in different concentrations of BBC/MST herbicides (**Supplementary Table S2**). The results showed that ‘Xiangling628S’ (XL628S) seedlings displayed significant phytotoxicity when seeds were soaked in a very low concentration of BBC (12.5 mg/L) (**Figure 1A**). The plants showed reduced plant height, yellowing and curling of leaves, withered leaf tips, thinner stems and bleached stems, although they were still able to grow. When the concentration of BBC was 37.5-112.5 mg/L, the leaves of XL628S were completely bleached and curled, the plants were thin and short, and had completely lost vitality, over time, the maternal materials died due to the toxic effects of the herbicides, thus, the purification efficiency of hybrid seed could reach 100%. In contrast, ‘Huazhan’ (HZ) and ‘Lingliangyou942’ (LLY942) plants were robust and the leaves were green and healthy, and it appeared that plant growth was not affected by the herbicides. The plant phenotypes indicated that 37.5-112.5 mg/L is a safe concentration range for improving the purity of hybrid rice seed with BBC. At a BBC concentration of 337.5 mg/L, LLY628S plants showed some phytotoxicity; leaf-tips were dry and curled, and the stem was bleached. As the herbicide concentration increased, the plant height became significantly shorter. At the highest concentration of 1,350 mg/L, plant height in HZ was significantly reduced due to phytotoxicity, and the leaves were dry and wilted. This preliminary experiment clearly showed that soaking seeds in BBC is effective for purification of two-line hybrid seed.

Relative to BBC, MST is more potent and has a more significant effect on plant growth (**Figure 1B**). At a concentration of 6.75 mg/L MST, XL628S plants were slightly bleached at germination, and when the treatment concentrations were 15.0 and 22.5 mg/L, LLY942 plants were slightly bleached on the fourth day after sowing even though the plants gradually recovered and turned green. HZ plants were unaffected at these concentrations, while the XL628S plants were totally bleached (**Figure 1B**). When the MST concentration was increased to 45.0 mg/L, the phenotypes of the three lines changed significantly; the XL628S and LLY942 plants were short, thin, and severely bleached, while plants of HZ were not completely bleached and showed weak growth vigor. Plants of all three lines were completely bleached and died when the treatment concentration was increased to 67.5 mg/L. Since the range of useful MST concentrations that are safe for seed treatment is too narrow and there is slight herbicide damage to the hybrids at the early growth stage, we determined that MST is not suitable for purifying two-line hybrid rice seeds.

In order to test whether the phenotypes of the two natural allelic variants of *HIS1* is consistent with the BBC soaking data, we selected XL628S(*his1-28bp*) and SE21S(*his1-T1510G*) (**Additional File 3**) for a second BBC seed soaking experiment using the same BBC concentration range (**Figure 1C**). The phenotypes showed that seedlings of both XL628S and SE21S were significantly stunted at the lowest concentration used (12.5 mg/L); the plants were shorter, with thinner bleached stems and white curled leaves. XL628S and SE21S seedlings were completely bleached and dead at BBC concentrations of 37.5-112.5 mg/L. Therefore, soaking seeds in BBC has the same effect on the lines carrying the two natural allelic variants of the *HIS1* gene. The germination rates of the experimental materials showed that the different concentrations of BBC and MST used in the soaking treatments had no significant effect on seed germination rates (**Figure 1D**).

### Treating rice seeds with BBC for 36-55 h is effective for hybrid seed purification in the two-line system

In practical applications, various uncertainties may adversely affect the hybrid seed purification effect. In order to test the effect of soaking time on the purification of two-line hybrid seeds, we selected a BBC concentration of 75.0 mg/L BBC for the experiment (**Figure 2A**). In the experimental group, XL628S seedlings died of bleaching caused by the herbicide. HZ plants were robust and healthy, with no difference in phenotype compared with the control due to the dominant herbicide resistance gene *HIS1*. LLY942 seedings grew normally after 36-55 h of BBC treatment, but there was some phytotoxicity, and the plants were slightly shorter when the soaking time increased to 56 h. Therefore, when using BBC seed treatment to improve the purity of two-line hybrid seeds, soaking the seeds for 36-55 h will not affect the normal growth of hybrids, which provides a favorable guarantee for practical production and application.

### β-triketone herbicides have no significant effects on the physiological and biochemical indexes of the rice hybrids

In our previous study, we initially showed the feasibility of BBC/MST spraying to improve hybrid purity in the two-line system based on phenotypic results (Lv et al., 2021). To further refine the effects of bTHs on the parental lines and hybrids, we examined the relevant physiological and biochemical indicators in the experimental materials treated with BBC/MST (**Figures 1A, B**, **3A**). The results demonstrated that the hybrid LLY942 (*HIS1/his1*) was significantly more resistant to bTHs than was XL628S (*his1-28bp*) in the range of 37.5-112.5 mg/L BBC by seed soaking. The seven growth indexes, including plant height, root length, stem thickness, shoot fresh/dry weight, and root fresh/dry weight were not significantly different from the untreated controls. When the BBC treatment concentration was increased to 337.5 mg/L, LLY942 began to suffer from herbicide toxicity, resulting in yellowed leaves, dwarfing, and significant reductions in various growth indicators. However, the male parent HZ was not poisoned by herbicides due to the presence of the dominant *HIS1* gene, and there were no statistically significant differences in growth phenotype and physiological indexes even at the higher treatment concentration (**Figure 3B**). Similar to soaking the seeds in BBC, LLY942 did not show any effects of the herbicide treatment when the BBC/MST sprays were within the safe concentration ranges of 50.0-600.0 and 10.0-100.0mg/L, respectively. The BBC/MST spray method of hybrid seed purification could reach 100% efficiency (**Figures 3A**). When the herbicide concentration increased, the hybrid showed bleaching and dwarfing due to herbicide toxicity, and all of the physiological indexes showed significant downward trends (**Figure 3C**).

Under normal circumstances, when the production and elimination of reactive oxygen species (ROS) in plants are in a state of dynamic equilibrium, the ROS levels will remain within a certain concentration range that does not negatively affect plant growth (Apel and Hirt, 2004; Dvořák et al., 2021; Sachdev et al., 2021). We selected the rice hybrid variety LLY942 using the three hybrid seed purification methods (BBC soaking, BBC/MST spraying) to examine the changes in chlorophyll content, H_2_O_2_ and MDA concentrations, and the enzyme activity of peroxidase (POD) (**Figure 3D**). The results showed that there was no significant change in chlorophyll a/b contents in the safe concentration range, while the chlorophyll content decreased significantly at higher herbicide concentrations, and high concentrations of MST applied by spraying had the greatest effect on chlorophyll content, with a 35.04% decrease. The chlorophyll contents decreased by 21.82% and 20.12% for LLY942 treated with the highest concentrations of BBC by soaking and BBC by spraying, respectively. The ROS H_2_O_2_ showed a significant upward trend with increasing herbicide concentration; at the same time, protective enzyme systems were activated, and the POD (peroxidase) activity increased significantly, which led to no significant change in oxidized lipids such as MDA in the safe range of herbicide concentrations for hybrid seed purification (Sachdev et al., 2021). However, high herbicide concentrations have a significant toxic effect on plants, which seriously destroys the membrane system, weakens the activities of plant protective enzyme systems, and eventually leads to an accumulation of ROS and oxidized lipids that hinders the normal growth and metabolism of cells. The data showed that MDA levels increased by 1.95-fold at the high concentration of MST in the spray treatment.

### bTHS-resistant material (Huazhan) may relieve the toxicity of bTHs through phenylalanine metabolism and phenylpropanoid biosynthesis pathway

To analyze how resistant materials relieve the toxicity of bTHs, Huazhan, LLY942, XL628S plants with or without BBC herbicide treatment was performed. A total of 299 up-regulated genes were identified in the resistant materials Huazhan, and was the largest number among the three materials. XL628s was seriously affected by herbicide poisoning, a total of 531 downregulated genes were differentially expressed in XL628S plants with treatment. (**Figure 4A**; p-value < 0.05 and |log2FC| >= 1). In order to verify the accuracy of transcriptome sequencing data, we selected several genes to carry out qRT-PCR. The qPCR data analysis results of up/down-regulated genes are consistent with the transcriptome sequencing results, indicating that the sequencing data are reliable. (**Supplementary Figure S3**) Venn of DEGs diagram analyses revealed an overlap of 28 genes in the three materials comparisons. 421 uniquely expresses genes were identified in the non-overlapping part of Huazhan, and 591 uniquely expresses genes in XL628S (**Figure 4B**). Those uniquely up-regulated DEGs of Huazhan were found to be enriched in phenylalanine metabolism and phenylpropanoid biosynthesis pathway, while up-regulated DEGs of XL628S were mainly enriched in starch and sucrose metabolism (**Figure 4C**; **Supplementary Figure S4**; p-value < 0.05). These results indicate that bTHs-resistant material (Huazhan) relieves the toxicity of bTHs through phenylalanine metabolism and phenylpropanoid biosynthesis pathway. These biological metabolic pathways can promote the synthesis of HPPD (Lin et al., 2019), (**Figure 4D**). Meanwhile, *HIS1* encodes an Fe (II)/2-oxoglutarate–dependent oxygenase that detoxifies bTHs by catalyzing their hydroxylation (Maeda et al., 2019). To further verify this conclusion, we carried out qRT–PCR to measure the expression levels of genes related to the phenylalanine metabolism and phenylpropanoid biosynthesis pathway. *OsPAL1*, a phenylalanine ammonia-lyase gene, the gene was up-regulated by 2.8 times in huazhan, while down-regulated by 1.55 times in XL628s (Tonnessen et al., 2015) It is associated with broad spectrum disease resistance, overexpression of *OsPAL1* in wild-type rice TP309 confers resistance to *M. oryzae* (Zhou et al., 2018). *OsAP211*(*ARAG1*), a rice DREB gene, is involved mainly in the ABA signalling and plant responses to abiotic stresses (Zhao et al., 2010), and *OsSGL* (*Stress_tolerance and Grain_length*) can enhance drought tolerance by regulating the expression of stress-responsive genes (Cui et al., 2016). The high expression of stress-resistant genes will lead to the increase of antioxidant enzyme activity in plants, and eliminate excess ROS in time, improve plant resistance. Such as *OsSGL*, the overexpressing plants accumulate higher levels of proline and soluble sugars but lower MDA contents under stress treatment. Thus, the hybrid LLY942 and Huazhan can be active within the range of safe application concentration (**Figure 3D**). These genes all increased significantly in HZ but decreased significantly in XL. The related up-regulated expression genes indicates that rice actively undergoes stress-resistant metabolism in the early stage of herbicide stress.

### β-triketone herbicides have no significant effect on grain yield of the two-line hybrids

**Figure 5:**
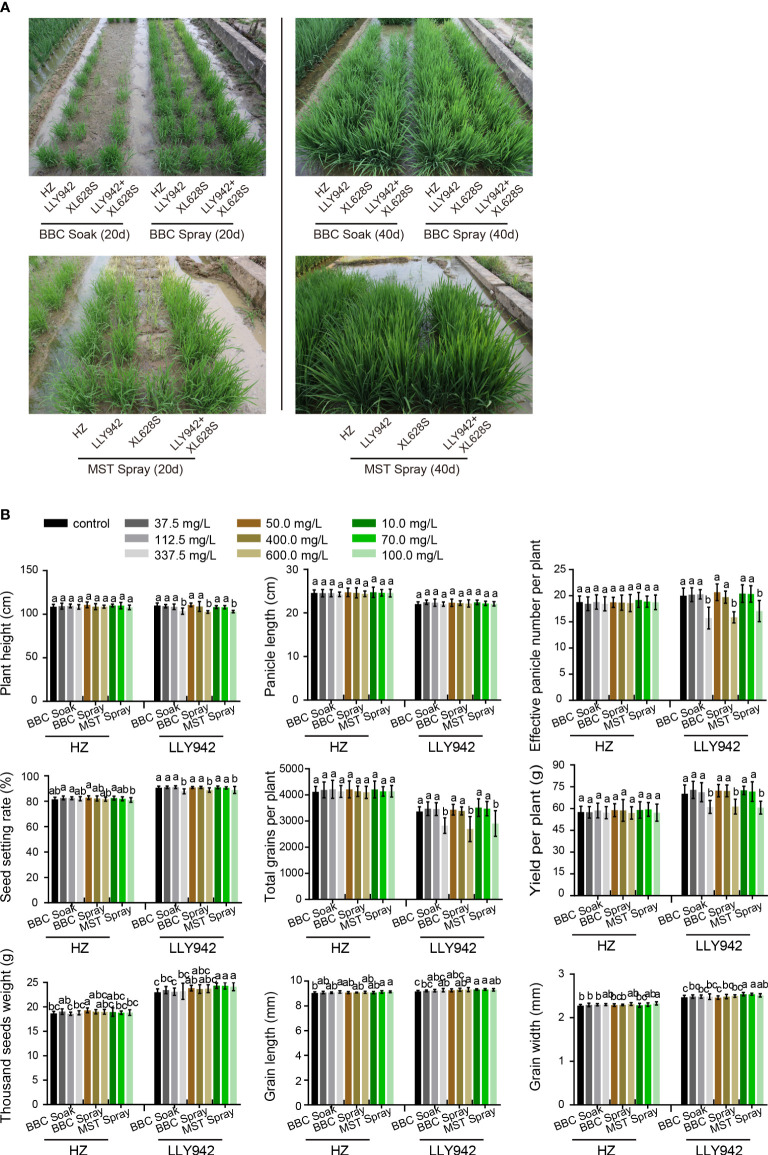
Plant phenotypes and agronomic characters of the rice lines HZ, LLY942, and XL628S treated with seed purification methods in a field experiment. **(A)** Phenotypes of the hybrid LLY942 and the parental lines HZ (male) and XL628S (female) treated with the four hybrid rice purification methods in the field. Seed soaking conditions were the same as used in the laboratory experiments. After sowing, the seedlings were sprayed with either BBC or MST when they reached the three-leaf stage. Phenotypic observations were made 20 and 40 days after sowing. **(B)** Effects of different bTH concentrations and the three purification methods on the agronomic characters of LLY942 and HZ. Data are the means ±SD of three biological replicates. Different letters above the bars indicate significant differences at *p* <0.05 (one-way ANOVA, Tukey’s test).

In order to test whether the herbicide concentrations found to be safe when used in the greenhouse for seed purity were feasible in the paddy field, we tested four seed purification methods: BBC soak, BBC spray, MST soak, and MST spray, with 11 concentrations used for each treatment group (**Supplementary Table S3**). The BBC/MST soak conditions for the field experiments were the same as those used in the laboratory experiments, while the BBC/MST sprays were applied at the three-rice seedling leaf stage at noon on a clear and cloudless day, and the field experiment was conducted at puddle-transplanted rice (**Figure 5A**).

The results clearly demonstrated that soaking the seeds in BBC solution had the most significant effect for the purification of two-line system seeds; the female parent XL628s was completely killed, while the hybrids showed healthy growth, and the purification rate reached 100% (**Figure 5A**). Secondly, the effect of BBC spraying at the three-leaf stage on the hybrid purification was remarkable. In the BBC concentration range 50.0-400.0 mg/L, plants of the sterile line XL628S were obviously killed by the herbicide, and the purification rate was 100% as well (**Figure 5A**). Compared with BBC, MST is much more potent. Spraying MST at extremely low concentrations (10.0-70.0 mg/L) killed P/TGMS line plants without affecting normal growth in the hybrids (**Figure 5A**). When the concentration was 200.0 mg/L, the plants of both LLY942 and HZ were seriously affected and bleached, and they turned white and died at a concentration of 300 mg/L MST. The results of the MST soaking experiment were unsatisfying: at germination, when the seedlings had just emerged from the soil, the sterile lines in the treatment group showed obvious bleaching, but as the plants grew to the three-leaf stage, they showed re-greening, and some even recovered and grew well. Therefore, we determined that improving seed purity during two-line hybrid seed production could not be achieved by soaking seeds in MST (**Figure 5A**).

To further explore whether herbicide treatment can affect important agronomic traits, we selected three experimental materials and treated them with low and moderate concentrations (in the safe purification concentration range) and a high concentration in the three applicable purification methods in the paddy field. The results showed that most of the agronomic characters of the male line HZ were barely affected even at the high concentration treatment in all three seed purification methods. Agronomic traits such as 1,000-seed weight, grain length, and grain width in HZ showed a slight increasing trend compared with the control (**Figure 5B**). Within the safe herbicide concentration range for seed purification, there were no significant negative effects on any agronomic trait in the two-line hybrid LLY942, but growth was seriously affected at high concentration; the plant height, yield per plant, effective panicle number, total grain number per panicle, and seed-setting rate decreased significantly, while the panicle length was not significantly different from the control. As we observed with HZ, the 1,000-seed weight, grain length, and grain width in LLY942 also showed a slight upward trend. Among the three treatments, MST spraying resulted in the most significant changes in agronomic traits such as 1,000-seed weight, grain length, and grain width, which increased by 1.06%, 0.86%, and 0.13% for HZ and 3.05%, 1.12%, and 1.66% for LLY942, respectively.

## Discussion

### β-triketone herbicides can be used to improve seed purity of two-line hybrid rice

As a crucial part of hybrid rice cultivation, the two-line system has had strong developmental momentum since it was first invented (Zheng et al., 2020; Wu et al., 2021). However, in the process of seed production, the P/TGMS lines suffer from a continued exposure to temperatures <23°C for three consecutive days during the sensitive fertility period, which leads to fertility restoration in the female parental line and then results in self-pollination, which affects the purity of hybrid seeds and can even cause seed production failures if the purity is <96% (Su et al., 2012; Wu et al., 2021). At present, the measures used to deal with the risks associated with seed production in two-line hybrid rice mainly include reducing the critical sterility-inducing temperature of P/TGMS lines, applying artificial temperature control with a cold water treatment system, using the original seeds of the sterile lines, selecting appropriate seed production bases and climatic conditions scientifically (the fertility sensitive safe period, heading and flowering safe period, and mature and harvest safe period), timely microscopic examination and isolated cultivation at the flowering stage, and chemical emasculation of the female parent (Lei et al., 2014; Hu et al., 2022; Yi et al., 2022). Active prevention can effectively improve the purity of two-line hybrid seeds and avoid seed production failures. However, the weather is not controlled by manpower, and the occurrence of abnormally low temperatures during the fertility sensitive period of the female parent will lead to the seed production failures, ultimately causing significant financial losses.

In view of the problem of reduced seed purity of two-line hybrid rice due to self-pollination of the female parent, we have made it clear that it can be solved based on the difference in tolerance to β-triketone herbicides between the female parent and the F_1_ hybrid. Our preliminary greenhouse experiments initially showed that spraying with BBC or MST can kill the female parent without affecting normal growth in the hybrids, and this can improve the purity of two-line hybrid rice (Maeda et al., 2019; Lv et al., 2021). The hybrid rice seed purity rate can reach 100% by soaking seeds in BBC at safe concentrations between 37.5 and 112.5 mg/L, and by spraying plants at the three-leaf stage with either BBC at 50.0-400.0 mg/L or with MST at 10.0-70.0 mg/L. Analysis of the physiological and biochemical characters in the greenhouse and agronomic characters in the paddy field experiment showed that the three methods of hybrid seed purification that can be used have no significant negative effects on the vegetative growth and yield of the rice hybrid and the male parental line. We also confirmed that soaking seeds in BBC at 75.0 mg/L for 36-55 h had no significant effect on the growth of hybrid rice, which strongly suggests that it is feasible to solve the problem of seed purity in the two-line hybrid rice using selective sensitivity to β-triketone herbicides. Besides *HIS1* detoxifies bTHs by catalyzing their hydroxylation, we also found that 299 up-regulated significant DEGs in the resistant material -Huazhan poisoned by BBC, were mainly enriched in phenylalanine metabolism and phenylpropanoid biosynthesis pathway, the content of HPPD was increased finally, it may also be exposed to the toxic effects of herbicides in this way.

In a previous study, we identified varieties which were not tolerant to β-triketone herbicides from more than 100 sterile two-line hybrid rice lines by analyzing the natural allelic variation in *HIS1*, a gene that gives broad-spectrum resistance to β-triketone herbicides in rice (Lv et al., 2021). The hybrid seed purification experimental methods are applicable to these bTH-sensitive sterile lines when the corresponding male parents of the two-line hybrid rice are tolerant of bTHs to achieve 100% hybrid seed purity. In addition, because the *his1* gene does not affect the agronomic characters of rice plants, the two-line sterile line and restorer line can be permanently maintained as herbicide-susceptible and herbicide-tolerant types, respectively, in future breeding programs, thereby assuring that the issue of seed purity in two-line hybrid rice can be addressed by the use of β-triketone herbicides in moderation.

### Exploring new approaches and application of two-line hybrid seed purification

Compared with using traditional paddy field cultivation to quantify hybrid seed purity, spraying bTHs can reduce the land required and the labor costs by treating seedlings in the field (Xiao, 2012). We demonstrated the feasibility of spraying herbicides to purify hybrid rice through experiments in the laboratory and in the field. In addition, we were the first to test the effects of soaking seeds in BBC, which can achieve hybrid seed purification before sowing. This approach saves on manpower and material resources needed for later spraying, and it is safer, more straightforward, and friendlier to the environment. In a previous study, a total of 631 indica rice accessions commonly used in Chinese rice breeding were tested, including 59 hybrid rice two-line restorer and 51 P/TGMS. The results of *HIS1* gene detection showed that *HIS1* genotype existed in 28 (47.46%) two-line restorers, meanwhile, 8 (15.7%) P/TGMS lines harbored the 28-bp deletion or T1510G natural allelic variations of *HIS1* (Lv et al., 2021), this means that the three purification methods of hybrid rice seeds can be widely used in two-line hybrid rice.

The bTH strategy can also be explored for use in direct-seeded rice. Direct seeding cultivation is the main way that rice is grown in Australia, the United States, the former Soviet Union, and some European countries. In Asia, South Korea, Japan, the Philippines and other countries, the land area devoted to direct seeding has shown an upward trend in recent years due to the advantages of reduced labor, a shorter growth period, higher grain yield (5% higher than PTR), and the fact that direct-seeding is more conducive to large-scale development (Luo et al., 2019). However, it is more susceptible to weed damage than TPR. It is necessary to carry out effective weed control options in DSR (Panneerselvam et al., 2020). Spraying herbicides at appropriate concentrations at the three-leaf stage of rice seedlings can not only kill weeds but also purify the hybrid seeds, thus “killing two birds with one stone”. Therefore, the bTH strategy has the potential to purify hybrid rice seeds in two-line seed production, whether by PTR or DSR.

At present, we are exploring using seed coatings in two-line hybrid seed purification which will make the system even easier to use. Seed coatings can be formulated to contain various medicinal ingredients that will dissolve and be released during seed soaking and can then be absorbed by the seeds. This method can also prevent diseases and control pests at the seedling stage, promoting growth and development and increasing crop yield (Ruan and Xue, 2002; Zhang et al., 2018; Cortés-Rojas et al., 2021). This is consistent with the BBC soaking principle. The appropriate concentration of BBC herbicide is mixed with the coating material and deposited on the surface of the hybrid seeds, which can not only improve hybrid seed purity, but can also effectively prevent diseases and pests in paddy fields, which is more conducive to commercial production and agricultural production.

### Other factors that affect the purification hybrid seeds

Throughout the experiments, we found that the effect of purifying hybrid seeds using the herbicide strategy was affected by several factors, including the different growth stages of the seedlings, the external temperature, and light intensity (Tasmin et al., 2018). The younger the rice seedlings are, the more susceptible they are to the toxic effects of herbicides. When the plant comes into contact with the selective bTHs, the herbicides can be absorbed by the leaves, stems, and roots and translocated throughout the plant in the vascular tissue. When external temperature and light intensity are high, plant metabolism is accelerated, making the herbicide act on the target site faster. This then inhibits the activity of HPPD, hinders the normal tocopherol and plastoquinone synthesis pathways and affects the biosynthesis of carotenoids, which ultimately affects photosynthesis in the plant, resulting in bleaching and death of the plants (Maeda et al., 2019). In the field experiment where we sprayed the bTHs, the temperature was as high as 34°C, which was much higher than the maximum temperature of 28°C in the greenhouse. Therefore, the optimal concentration range of BBC for spray application in the field was reduced slightly to 50.0-400.0 mg/L, and 10.0-70.0 mg/L for spraying MST in the field.

When MST was used to soak the seeds for hybrid seed purification, the female parental line was completely bleached due to the toxic effects of the herbicide at germination; however, the plants gradually recovered, turned green, and resumed growth. This phenomenon may be caused by the shorter half-life of MST compared with BBC. For example, the half-life of MST in Beijing and Shandong soils is 4.31 days and 1.80 days, respectively, and the degradation rate of MST in the germinating stage after sowing is accelerated due to direct contact with the soil and water on the surface. Therefore, MST fails quickly after seed soaking to sowing, and some seedlings ultimately recover their ability to grow (Sun et al., 2013; Su et al., 2017). In addition, although the female parental line can be killed in the greenhouse by 15.0 mg/L MST, the safe concentration range for two-line hybrid purification is too narrow to be suitable for field application.

## Conclusions

We found that we can effectively achieve 100% seed purity in the two-line hybrid rice system in which the offspring of female parent self-pollination is mixed with the true hybrid seed by soaking the seeds in BBC at 37.5-112.5 mg/L or by spraying rice seedlings at the three-leaf stage with BBC at 50.0-400.0 mg/L or with MST at 10.0-70.0 mg/L. Meanwhile bTHs-resistant material (Huazhan) may relieve the toxicity of bTHs through phenylalanine metabolism and phenylpropanoid biosynthesis pathway. The effect of the herbicide is remarkable and the operation of the system is straightforward, which has a significant application value in agricultural production.

## Data availability statement

The original contributions presented in the study are publicly available. This data can be found here: https://ncbi.nlm.nih.gov/sra/PRJNA974916.

## Ethics statement

The varieties in the current research are not threatened species. The authors declare that we comply with the IUCN Policy Statement on Research Involving Species at Risk of Extinction. Experimental research and field studies on plants comply with relevant institutional, national, and international guidelines and legislation.

## Author contributions

BZ and QL conceived and supervised this research. XZ performed most of the experiments and wrote the manuscript. QW, GF and BM conducted the field experiments, performed phenotyping. YS and LT conducted the green-house experiments, and analyzed the data. All authors read and approved the final manuscript.

## Supplementary material

Supplementary Table 1 The primers used for quantitative real-time PCR.

Supplementary Table 2 Seeds were treated by soaking in different concentrations of BBC/MST herbicides.

Supplementary Table 3 Four hybrid seed purification methods using different herbicide concentration ranges.

Additional File 1
*HIS1* gene sequence of Huazhan.

Additional File 2
*HIS1* gene sequence of Xiangling628S.

Additional File 3
*HIS1* gene sequence of SE21S.
