# Supplementary figures and images for: Utilizing differences in bTH tolerance between the parents of two-line hybrid rice to improve the purity of hybrid rice seed
Source: Front Plant Sci. 2023 Aug 3;14:1217893. doi: 10.3389/fpls.2023.1217893 (PMC10435883; doi:10.3389/fpls.2023.1217893)

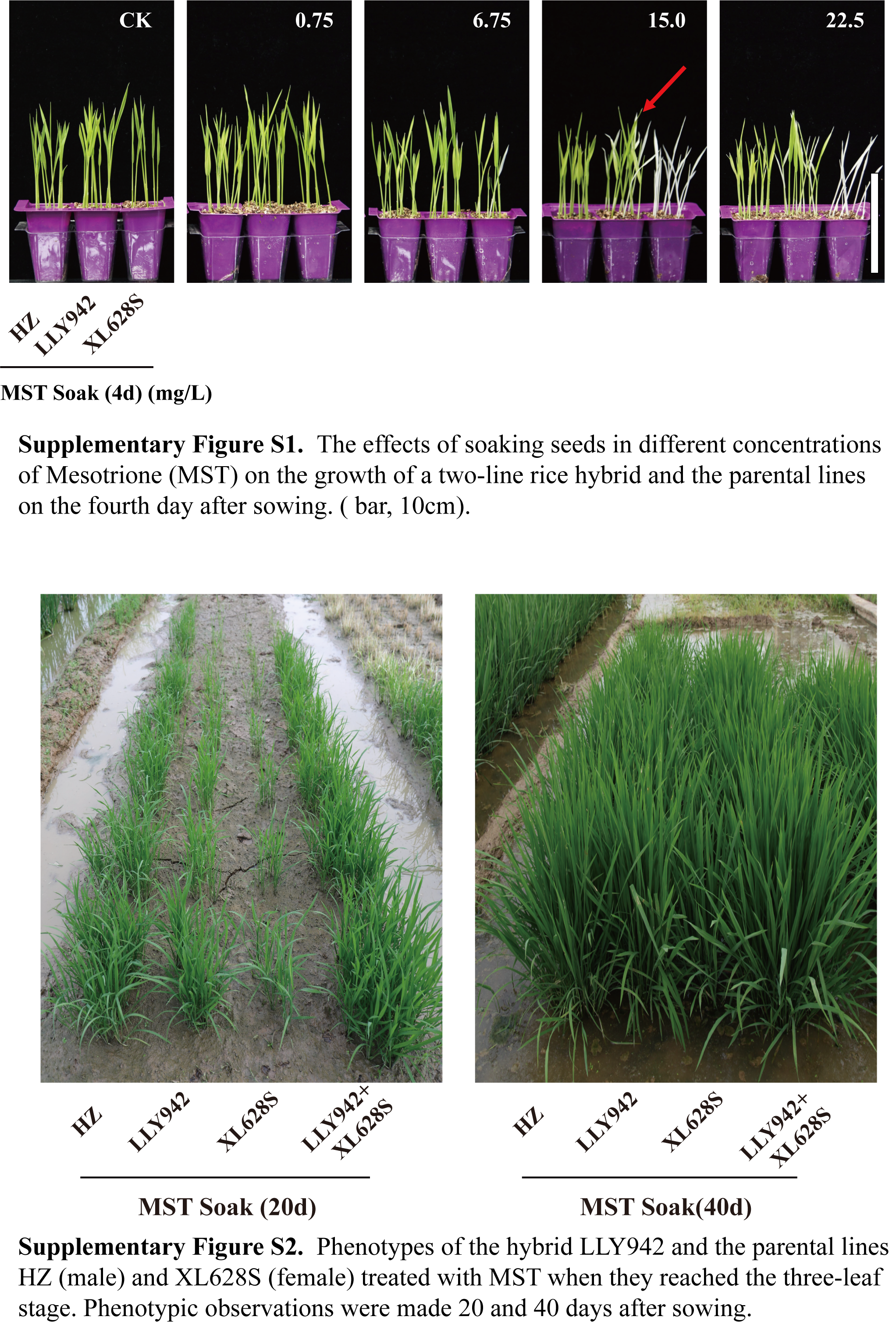

Supplement: Supplementary file 1 [file Image_1.tif]

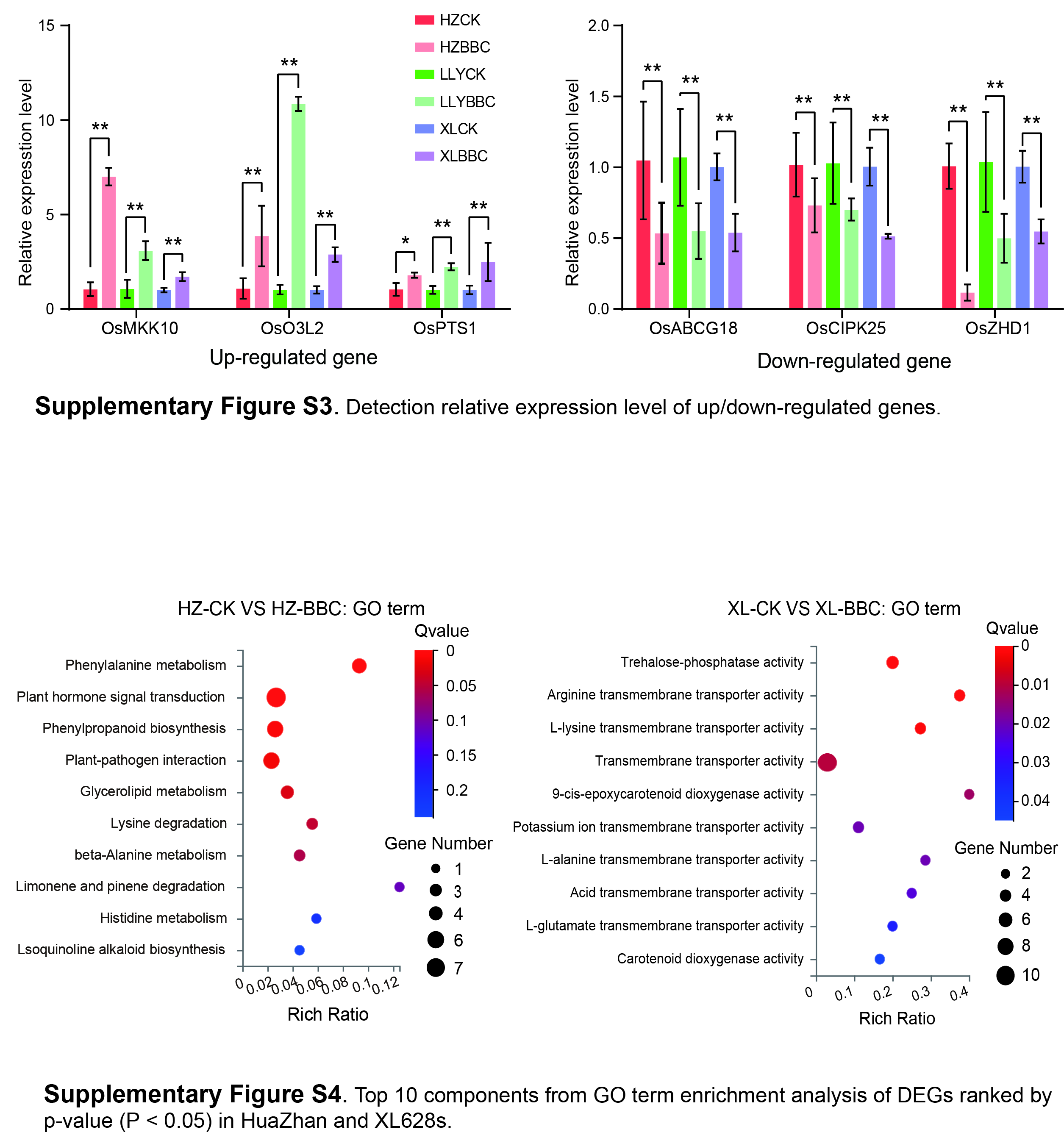

Supplement: Supplementary file 2 [file Image_2.tif]
