# Supplementary material for: Utilizing differences in bTH tolerance between the parents of two-line hybrid rice to improve the purity of hybrid rice seed
Source: Front Plant Sci. 2023 Aug 3;14:1217893. doi: 10.3389/fpls.2023.1217893 (PMC10435883; doi:10.3389/fpls.2023.1217893)
Supplement: Supplementary Table 1 — The primers used for quantitative real-time PCR. [file DataSheet_1.zip › Supplementary Material/Additional File 1.DOCX]

>HuaZhan

ATGGCTGATGAGTCATGGAGGGCGCCGGCGATAGTGCAAGAGCTGGCGGCAGCCGGCGTCGAGGAGCCGCCGAGCCGATA

CCTGCTACGGGAGAAAGACCGTTCTGACGTCAAGCTGGTCGCCGCCGAGCTGCCGGAGCCCCTCCCCGTCGTTGATCTCA

GCCGGCTAGATGGTGCCGAGGAGGCCACCAAGCTCAGGGTGGCTCTGCAGAATTGGGGCTTCTTCCTGGTCAGCTTCTAA

CAAGTGATTCTACTTTGCTTCATAAAAAAGACTTGCTCATTTGTATTCATTTCTCCAATTTGTGTGGTTGTGTGTGATCC

AGCTTACCAACCATGGAGTAGAAGCCTCTCTGATGGACAGCGTGATGAACTTGTCGAGAGAGTTTTTCAACCAACCAATC

GAACGGAAGCAAAAATTCAGCAACTTGATCGATGGCAAGAACTTCCAGATTCAAGGGTATGGAACTGACCGGGTGGTTAC

CCAAGATCAGATCCTGGACTGGTCTGATCGGTTGCATCTCAGAGTTGAACCCAAGGAGGAGCAAGATCTTGCCTTCTGGC

CTGACCATCCTGAATCTTTCAGGTCACCTACTCACCTCACATTGATCGATGTTTTACTTTCCAGTTTCCACACGTCTGAA

TTTCTTTCTCTTTTGTTTTTTCTTTTTTTGCAAAAGATAGTGTTTCTTACTGTTCATATATTACTTACAAAGTAACAAGG

ATTGTTGTCTGAATTCAGAAAGTACAACTTGACGATGTATCAAGAAATGGTTTTGCTGAGCTATTTGAGAGCCTTTTCTT

CTGCAGGGATGTTCTGAACAAGTATGCATCAGGAACCAAAAGAATTAGAGACGATATCATTCAGGCTATGGCCAAGCTTC

TTGAGCTTGATGAGGATTACTTCTTGGACCGACTCAACGAAGCTCCTGCATTTGCAAGATTCAACTACTACCCTCCCTGT

CCAAGGCCTGACCTTGTGTTCGGCATCAGGCCTCACTCCGACGGCACCCTCTTGACGATTCTTCTCGTCGACAAAGATGT

CAGTGGCCTGCAAATTCAGAGGGATGGCAAGTGGTCCAACGTTGAGGCAACTCCTCACACATTGCTGATCAACTTAGGTG

ACACCATGGAGGTAATTGCCTTATTGGCATCCAACATCCAAAATACTTACTCTCTGAATCTGCAGACATCCAGTACTTCC

TCCGTTTCATATTATAAGACTTTCTAGCATTGCCCATATTCATATATATGTTAATGAATCTGGACACATATATGTCTAGA

TTCATTAATATATATATATATATAAATGTTGTTAATGTGTCTAGATTCATTAACATATATATGAATGTGGGCAATGCTAA

AAAGTCTTATGACCTGAAACGAAGGTAGTATATCTGATGATAAATCCAGGGTTTCTGAATGCTGATGCATATTTGGATGC

AACATTTCTGTGCAGGTAATGTGCAATGGCATCTTCAGGAGCCCGGTGCACAGGGTGGTGACAAACGCCGAGAAGGAGAG

GATCTCCCTGGCCATGTTATACAGCGTGAACGATGAGAAAGACATTGAGCCGGCGGCTGGTTTGCTGGATGAGAATCGGC

CTGCAAGATACAGGAAAGTGAGCGTCGAAGAGTTCAGGGCCGGGATCTTTGGAAAATTCTCTCGAGGAGAGAGGTACATC

GACTCCCTGAGGATCTGA
