# Supplementary material for: Utilizing differences in bTH tolerance between the parents of two-line hybrid rice to improve the purity of hybrid rice seed
Source: Front Plant Sci. 2023 Aug 3;14:1217893. doi: 10.3389/fpls.2023.1217893 (PMC10435883; doi:10.3389/fpls.2023.1217893)
Supplement: Supplementary Table 1 — The primers used for quantitative real-time PCR. [file DataSheet_1.zip › Supplementary Material/Tables S2 and S3.DOCX]

**Supplemental Table S2: Seeds were treated by soaking in different concentrations of BBC/MST herbicides**

| **Seeds were treated by soaking in different concentrations of BBC/MST herbicides** | | | | | | | | |
| --- | --- | --- | --- | --- | --- | --- | --- | --- |
| **Concentration（mg/L) Herbicide** | **CG** | **TG-1** | **TG-2** | **TG-3** | **TG-4** | **TG-5** | **TG-6** |  |
| **Benzobicyclon (BBC)** | **0** | **12.5** | **37.5** | **112.5** | **337.5** | **1012.5** | **1350.0** |  |
| **Mesotrione (MST)** | **0** | **0.75** | **6.75** | **15.0** | **22.5** | **45.0** | **67.5** |  |
| CG:control group; TG:treatment group | | | | | | | | |

**Supplemental Table S3: Four hybrid seed purification methods using different herbicide concentration ranges**

| **Four hybrid seed purification methods using different herbicide concentration ranges** | | | | | | | | | | | | | |
| --- | --- | --- | --- | --- | --- | --- | --- | --- | --- | --- | --- | --- | --- |
| **Concentration（mg/L) Method** | **CG** | **TG-1** | **TG-2** | **TG-3** | **TG-4** | **TG-5** | **TG-6** | **TG-7** | **TG-8** | **TG-9** | **TG-10** | **TG-11** |  |
| **Benzobicyclon (BBC) Soak** | 0 | 75.0 | 150.0 | 225.0 | 300.0 | 375.0 | 450.0 | 600.0 | 900.0 | 1200.0 | 1500.0 | / |  |
| **Benzobicyclon (BBC) Spray** | 0 | 12.5 | 25.0 | 50.0 | 200.0 | 400.0 | 600.0 | 750.0 | 900.0 | 1200.0 | 1500.0 | 1800.0 |  |
| **Mesotrione (MST) Spray** | 0 | 10.0 | 30.0 | 70.0 | 100.0 | 200.0 | 300.0 | 400.0 | 500.0 | 600.0 | 700.0 | 800.0 |  |
| **Mesotrione (MST) Soak** | 0 | 25.0 | 50.0 | 100.0 | 200.0 | 300.0 | 400.0 | 600.0 | 800.0 | 1000.0 | 1200.0 | / |  |
| CG:control group; TG:treatment group | | | | | | | | | | | | | |
